# Pasteurella Cerebral Mycotic Aneurysm: A Case Report and Review of the Literature

**DOI:** 10.7759/cureus.15312

**Published:** 2021-05-29

**Authors:** Vidya S Kollu, Lennox Archibald, Matthew Edwards, Jennifer W Janelle, Kyung W Hong, Gautam Kalyatanda

**Affiliations:** 1 Department of Infectious Diseases and Global Medicine, College of Medicine, University of Florida, Gainesville, USA; 2 Department of Infectious Diseases, Case Western Reserve University School of Medicine, Cleveland, USA

**Keywords:** mycotic aneurysm, infective endocarditis, pasteurella infection, intracranial hemorrhage, cerebral aneurysm

## Abstract

*Pasteurella *species (spp.) are pleomorphic, Gram-negative, facultatively anaerobic bacilli commonly found in the upper respiratory tract and oral cavities of wild and domesticated animals such as dogs and cats. *Pasteurella* spp. infections in humans are typically caused by animal bites or scratches, or other inadvertent exposure of an open skin lesion to oral secretions of the animal. While skin and soft tissue infections are relatively common, respiratory infections, endocarditis, osteomyelitis, meningitis, and mycotic aneurysms have also been documented. To date, nine cases of mycotic aneurysms caused by *Pasteurella* spp. have been reported. However, only one of those cases has involved a cerebral mycotic aneurysm, and it had a fatal outcome. This report describes a successfully managed *Pasteurella* cerebral mycotic aneurysm that had occurred as a complication of underlying mitral valve endocarditis.

## Introduction

*Pasteurella* species (spp.) are pleomorphic, facultatively anaerobic, Gram-negative bacilli, which were first isolated by Louis Pasteur in 1880. These bacteria are commonly found in the upper respiratory tract and oral cavities of wild and domesticated animals, including dogs and cats [1]. *Pasteurella* spp. typically cause infections among humans following transmission via an animal bite, scratch, or lick, or by inhalation of infected secretions [2]. Also, rare cases of human-to-human and vertical transmission have also been described [3]. *Pasteurella* spp. commonly cause infection on the skin and soft tissues [2]. Rare invasive infections due to *Pasteurella* spp., including respiratory infections, endocarditis, osteomyelitis, meningitis, and mycotic aneurysms, arise almost exclusively among susceptible individuals including the immunocompromised, elderly, and neonates [2]. In this report, we present a rare case of a ruptured cerebral mycotic aneurysm complicating prosthetic valve endocarditis caused by *Pasteurella *spp. To the best of our knowledge, this is the second reported case of *Pasteurella* cerebral mycotic aneurysm and the first one with a successful outcome.

## Case presentation

A 57-year-old woman with a past medical history of atrial fibrillation, hypertension, cervical spine injury with C5-C6 fusion, congestive heart failure, and mitral valve endocarditis status post mechanical mitral valve replacement seven years ago presented with sudden onset of left-sided weakness and an inability to stand. These symptoms were associated with a two-week history of fever, chills, myalgia, and anorexia. She denied headache, nausea, vomiting, chest pain, or shortness of breath. On examination, the patient’s temperature was 37 °C (98.6 °F), pulse rate was 73 beats per minute, respiratory rate was 19 breaths per minute, and blood pressure was 166/63 mmHg. She was saturating at 98% on ambient room air. The cardiovascular exam revealed a grade 2/6 systolic murmur and grade 3 pedal edema. The oral exam showed absent dentition, and the musculoskeletal exam revealed bilateral palmar Dupuytren’s contractures and multiple scratch wounds on the lower extremities with no evidence of cellulitis or lymphangitis. The central nervous system exam revealed left-sided hemiparesis. The rest of the physical examination was unremarkable. Her social history was significant for a 25 pack-year history of smoking (she had quit 15 years ago), occasional alcohol use, and no illicit drug use. She admitted to sustaining scratch injuries while playing with her two dogs; further questioning revealed that the dogs had subsequently licked the wounds. 

**Figure 1:**
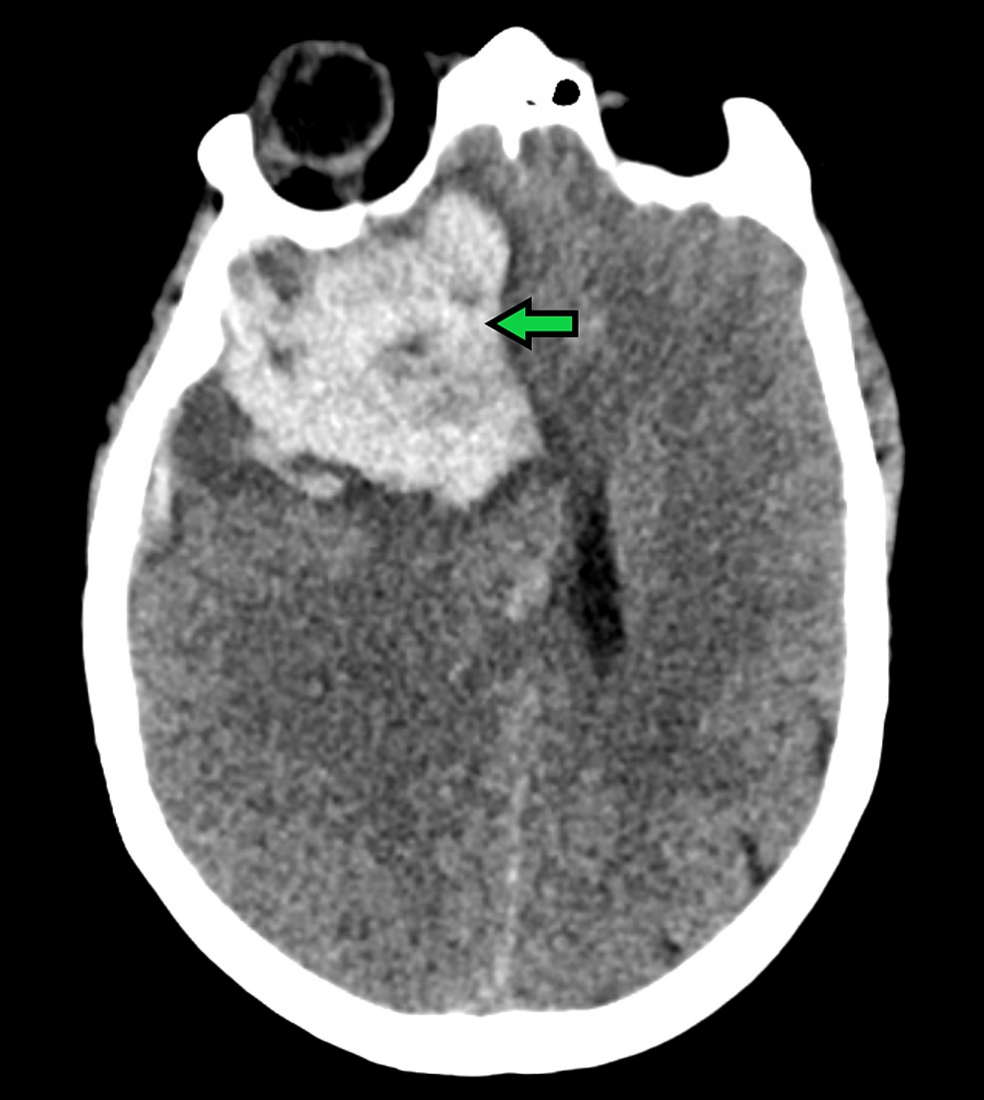
CT of the head showing a large right frontal hematoma with midline shift (arrow) CT: computed tomography

Laboratory testing showed a normal white blood cell count of 5.2 x 10^9^/L (normal range: 4.0-10.0 x 10^9^/L), low hemoglobin of 10.4 gm/dL (normal range: 12.0-16.0 gm/dL), and hematocrit of 31.5% (normal range: 35-45%). The basic metabolic panel was within normal limits. Chest radiography revealed bibasilar subsegmental atelectasis with a minimal left pleural effusion. CT of the head revealed a large right frontal hematoma with midline shift (Figure 1), and CT angiogram indicated active extravasation of contrast.

The patient underwent emergent craniotomy with hematoma evacuation. A mycotic aneurysm with cerebritis was found intraoperatively, and the aneurysm was clipped. An external ventricular device (EVD) was placed. She remained intubated and was started on broad-spectrum antimicrobial therapy, including intravenous (IV) vancomycin and cefepime. Blood cultures yielded growth of *Pasteurella* spp., which were susceptible to all antibiotics tested. Minimum inhibitory concentrations (MIC) were as shown in Table 1.

**Table 1:** Antibiotic MIC and sensitivities of the cultured Pasteurella MIC: minimum inhibitory concentration

Antibiotic	MIC, μg/mL	MIC interpretation
Ampicillin	0.25	Susceptible
Benzylpenicillin	0.12	Susceptible
Ceftriaxone	≤0.03	Susceptible
Chloramphenicol	≤0.5	Susceptible
Erythromycin	Not reported	Susceptible
Levofloxacin	≤0.03	Susceptible
Tetracycline	0.5	Susceptible
Trimethoprim-sulfamethoxazole	≤0.06	Susceptible

**Figure 2:**
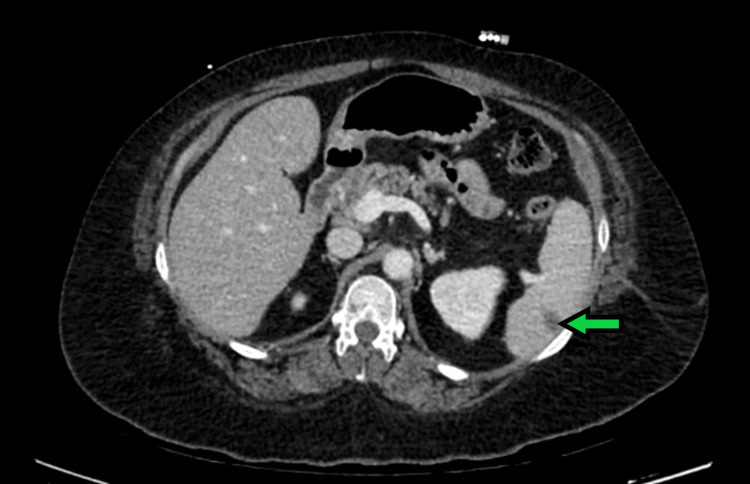
CT of the abdomen showing a wedge-shaped infarct of the spleen (arrow) CT: computed tomography

Subsequently, her therapeutic regimen was switched to penicillin G 24 million units IV by continuous infusion every 24 hours. Because of the presence of a mycotic aneurysm and the fact that the patient also had a prosthetic mechanical mitral valve, CT imaging of the chest and abdomen was carried out, which showed wedge-shaped hypodense areas in the spleen (Figure 2).

Transesophageal echocardiogram (TEE) revealed 1-cm mobile vegetation on the mechanical mitral valve. Repeat blood cultures 48 hours after the initiation of antibiotics were negative for bacterial and fungal growth. A week postoperatively, the EVD was removed, and a repeat CT angiogram of the brain showed no evidence of residual aneurysm or arteriovenous malformation. A repeat TEE two weeks later revealed that the prosthetic mitral valve vegetation was increasing in size (1.2 cm) compared to the vegetation seen on the previous TEE. Hence, the patient underwent redo sternotomy, removal of the in-situ mechanical mitral prosthetic valve, and implantation of a mitral bioprosthetic valve. The mechanical mitral prosthetic valve that was surgically removed was negative for bacterial and fungal growth. Although the patient`s postoperative course was complicated by atrial fibrillation, her condition gradually improved, and she was discharged to a rehabilitation center to complete a six-week course of IV penicillin G 24 million units administered via continuous infusion every 24 hours, counting from the day of valve replacement. The patient was found to be doing well at her four-week follow-up appointment.

## Discussion

Infective endocarditis usually involves the endocardial surface of the heart, and typically affects one or more heart valves. Prosthetic heart valves are particularly susceptible to endocarditis, as alterations to the flow characteristics of the valve encourage the deposition of microthrombi, to which pathogenic organisms adhere [4]. In cases of prosthetic valve endocarditis, the aortic valve is most frequently involved (69.1%), followed by the mitral valve (50.4%) [4]. Patients with infective endocarditis may present with one or more of a variety of symptoms (e.g., fever, chills, rigors, night sweats, malaise or fatigue, or embolic phenomena) and/or clinical manifestations, including new or changed murmur, focal neurological signs, splinter hemorrhages, Osler's nodes, Janeway lesions, Roth spots, or splenomegaly [5]. Our patient with prosthetic mitral valve endocarditis had, in addition to a typical presentation that included fever and a splenic infarct, the unusual complication of an acute intracranial hemorrhage due to the rupture of a cerebral mycotic aneurysm.

Cerebral mycotic aneurysms are rare, life-threatening lesions that develop due to the deterioration of intracranial or extracranial arterial walls caused by microbial infection [4]. One review has found that up to 65% of mycotic aneurysms originate from hematogenous spread from left-sided bacterial endocarditis, and further reported that 3-10% of patients with infective endocarditis develop mycotic aneurysms [6]. The friable cardiac vegetations of infective endocarditis produce septic emboli that may lodge at distal branch points of the middle cerebral artery, thereby occluding the vasa vasorum and leading to infarction, infection, and aneurysm formation [6]. Mycotic aneurysms may result from fungal or viral infections, though the majority of them originate from bacteria, particularly *Staphylococcus* spp. and *Streptococcus* spp. [3]. *Pasteurella* spp. have been described as a cause of mycotic aneurysms in only nine previous cases (Table 2) [7-15], one of which involved a cerebral mycotic aneurysm [7]. This was reported in a 17-year-old male with amoxicillin-treated mitral valve endocarditis, whose mycotic aneurysm was not recognized until autopsy.

**Table 2:** Summary of Pasteurella mycotic aneurysm cases M: male; F: female; EVAR: endovascular aortic repair; CVA: cerebrovascular accident

Author (year)	Patient age, Sex	Epidemiology	Location of aneurysm	Associated Pasteurella infection	Antimicrobials	Surgical procedure	Outcome
Pestana (1974) [8]	61, M	No bites described; dogs and cats at home	Abdominal aorta	Left elbow and right first middle carpal septic joints, bacteremia	Penicillin G	Open surgical repair	Died on the operating table
Goldstein et al. (1986) [9]	61, F	Cat bite	Thoracic aorta	Not described	Not described	Open surgical repair	Not described
Thamlikitkul and Sangruchi (1990) [7]	17, M	Denied animal bite or contact	Cerebral aneurysm diagnosed on autopsy	Mitral valve endocarditis, bacteremia	Ampicillin	None	Died from CVA
Balestra (2000) [10]	54, M	Dog lick, psoriatic lesions	Thoracic/abdominal aorta	Bacteremia	Amoxicillin, gentamicin	EVAR performed one year later	Alive 2 years after diagnosis
Koelemay (2009) [11]	64, M	Denied bites; cats at home	Abdominal aorta	Right leg cellulitis, no bacteremia described	Cefotaxime	Open surgical repair	Alive 1 year after surgery
Cho et al. (2016) [12]	68, M	Cat bite	Abdominal aorta	Cellulitis of right thumb, bacteremia	Piperacillin-tazobactam	Open surgical repair	Died on day 13 post-surgery from septic shock
Shalan et al. (2017) [13]	69, F	Cat bite	Abdominal aorta	Bacteremia	Penicillin G	EVAR followed by open surgical procedure	Alive 8 months after surgery
Kano et al. (2020) [14]	61, M	Dog lick	Abdominal aorta and aortic arch	Bacteremia	Ampicillin	Open surgical repair	Alive 1 year after surgery
Jeng et al. (2020) [15]	61, M	Dog bite	Abdominal aneurysm	Bacteremia	Not described	Open surgical repair	Alive 18 months after surgery
Our Case (2021)	57, F	Dog lick, scratch injuries	Cerebral aneurysm	Mitral valve endocarditis, bacteremia	Penicillin G	Open surgical clipping	Doing well 4 weeks after surgery

Cerebral mycotic aneurysms are associated with high morbidity and mortality, approaching rates as high as 80% in ruptured and 30% in unruptured cases [16]. There are no standard treatment guidelines for cerebral mycotic aneurysms due to the rarity of the disease and the lack of randomized controlled trials; the management generally involves antimicrobial therapy for unruptured aneurysms and combined treatment involving antimicrobials and surgery for ruptures [6]. Surgical repair of a ruptured aneurysm may entail aggressive debridement of the infected tissue and reconstruction of the vasculature [6]. The choice of antimicrobials is guided by the susceptibilities of the infecting organism and the agent’s capacity to penetrate the blood-brain barrier (BBB).

Our patient survived the catastrophic complication of a ruptured cerebral mycotic aneurysm thanks to the rapid recognition of the event, emergent surgical intervention, and initiation of broad-spectrum antimicrobial agents with excellent BBB penetration. *Pasteurella* spp. are invariably susceptible to penicillins, which can cross the BBB to maintain therapeutic concentrations in the central nervous system. The only other reported case of *Pasteurella *spp. cerebral mycotic aneurysm resulted in a fatal outcome, most probably due to a delay in diagnosis rather than in the commencement of treatment [7]. Our case underscores the utmost importance of detailed history and physical examination in the current era of high technology, and it highlights the value of prompt surgical and antimicrobial management to achieve optimal outcomes for patients with mycotic aneurysms in the cerebral vasculature.

## Conclusions

*Pasteurella* spp. infection should be considered in the differential diagnosis when investigating the pathogenesis of mycotic aneurysm in patients who had initially sustained bites, other trauma, or inadvertent exposure of wounds, scratches, or ulcers to oral secretions of domestic animals. A joint surgical and infectious disease approach with prompt commencement of antimicrobial therapy is crucial in the management of mycotic aneurysms for achieving favorable outcomes. Delays in the presentation by patients or in diagnosis by healthcare providers may result in substantial morbidity and mortality. Finally, choosing an antimicrobial agent with excellent BBB penetration is essential for the successful treatment of mycotic cerebral aneurysms.
